# Juvenile-onset multifocal osteonecrosis in systemic lupus erythematosus: A case report

**DOI:** 10.1097/MD.0000000000024031

**Published:** 2021-01-15

**Authors:** Wenyuan Jin, Xinghui Yang, Meiping Lu

**Affiliations:** a Department of Developmental Behavioral Pediatrics; b Department of Radiology; c Department of Rheumatology Immunology and Allergy, Children's Hospital, Zhejiang University School of Medicine, National Clinical Research Center for Child Health, No. 57, Zhugan Lane, Hangzhou 310003, China.

**Keywords:** juvenile-onset, osteonecrosis, pathogenesis, systemic lupus erythematosus, treatment

## Abstract

**Rationale:**

Osteonecrosis (ON) is a devastating illness that leads to bone ischemia and potential joint destruction. Systemic lupus erythematosus (SLE) is a chronic, autoimmune disease, with multi-system involvement which is closely associated with occurrence of ON. Multifocal ON, with an estimated morbidity of 3% in SLE patients, is extremely rare in juvenile subjects.

**Patient concerns:**

A 13.3-year-old female SLE patient was admitted to hospital 20 months following the SLE diagnosis because of a sudden aggravation of sore knees. She suffered from double knee joint pain and her left knee joint showed typical signs of inflammation including redness, swelling, heat, and pain.

**Diagnoses:**

The SLE patient was diagnosed with multifocal ON of her knee joint based on magnetic resonance imaging findings of bone destruction and osteoproliferation at the bilateral distal femur and proximal tibia.

**Interventions:**

The patient received high-dose methylprednisolone and intravenous cyclophosphamide pulse therapies for controlling active lupus and nephritis. Oral calcitriol and dipyridamole were administered to alleviate knee pain and inhibit thrombi formation, thereby suppressing ON progress.

**Outcomes:**

Three weeks following the treatment, the swelling in patient's left knee subsided. Her self-reporting pain score decreased from 9 to 4 and walking time increased from 45minutes to 90minutes per day. Nearly 5 weeks later, the pain in bilateral knee joints disappeared and the patient could walk without difficulties.

**Lessons:**

This patient is the youngest SLE patient who developed multifocal ON based on the reported literature. It suggests that ON can occur in young SLE patients. A combination of internal and external risk factors can promote the development of ON. The balanced approach to the application of corticosteroids and immunosuppressors in the treatment of SLE and prevention of ON is a challenging problem that deserves further exploration.

## 1 Introduction

Systemic lupus erythematosus (SLE) is a chronic, autoimmune disease with multi-system involvement, and unclear pathogenesis. Osteonecrosis (ON) is one of the common complications of SLE that manifests with joint pain, bone destruction, and walking difficulties. Prevalence of ON in SLE ranges from 3% to 44%.^[1–4]^ Compared with adolescent and adult SLE patients, pediatric patients have significantly lower rates of ON.^[2]^ The most frequently affected site in ON is the femoral head, followed by knees, hips, shoulders, and ankles. Elbow, wrist, and foot involvement have also been reported, but rare in SLE patients.^[4]^ Multifocal ON, which has been defined as occurrence in 3 or more anatomic sites have osteonecrosis lesions, but is not frequent in SLE and has a morbidity of approximately 3%.^[5]^ Magnetic resonance imaging (MRI) is regarded as a gold standard in the diagnosis of ON and it helps to detect both symptomatic and silent ON.^[6]^ Several mechanisms participate in the formation of ON in SLE. Fat accumulation in the bone increases intramedullary pressure and impairs endothelial cells, resulting in capillary rarefaction, disturbance of coagulation-fibrinolysis system, and thrombi formation.^[7,8]^ Meanwhile, decreased expression of vascular endothelial growth factor (VEGF) suppresses angiogenesis and aggravates interruption of blood supply and lack of oxygen in bone tissues.^[8,9]^ In addition, increased apoptosis of osteoblasts and osteocytes, prolonged lifespan of osteoclasts, and destroyed bone repair system also play an important role in the development of ON.^[10]^

## 2 Case presentation

A 11.6-year-old Chinese girl was diagnosed with SLE in October 2017. Initially, she presented with fever, malar butterfly erythema, vasculitis, hemolytic anemia, heavy proteinuria, C3 and C4 hypocomplementemia, hepatic function damage, positive direct Coombs’ test, positive antinuclear antibodies (ANA), anti-dsDNA, anti-Sm, anti-SSA, and anti-SSB antibodies. During the first 2 months after diagnosis (1st to 3th hospitalizations), she received high-dose methylprednisolone pulse therapies (0.5 g daily for 2 days) twice and intravenous cyclophosphamide pulse therapies (400 mg every time for a cumulative dose of 1.2 g) thrice. Moreover, hydroxychloroquine (0.2 g daily), mycophenolate mofetil (0.75 g daily), and tacrolimus (3 mg daily) were administered during the subsequent treatments. The patient experienced a relapse with appearance of a new malar butterfly erythema and erythrocyte sedimentation rate (ESR) increased to >140 mm/hour, accompanied by ANA titer increase to 1:1000 at the 4th hospitalization (Table 1). Through adjustments of corticosteriods dosage and combination of immunosuppressors, the patient's active lupus was gradually controlled and her clinical manifestations improved. At the 7th hospitalization, her ANA titer, ESR, 24-hour urinary protein quantity, and system lupus erythematosus disease activity index (SLEDAI) all significantly decreased and complement C3 significantly increased (Table 1).

**Table 1:** Clinical features of the systemic lupus erythematosus patient with osteonecrosis during every hospitalization.

Hospitalizations	ANA titer	Anti-dsDNA	ESR (mm/h)	24 h urinary protein (mg)	Complement C3 (g/L)	SLEDAI
1	+1:100	Positive	92	8603.1	0.22	19
2	+1:320	Negative	105	2417.8	0.37	15
3	+1:320	Negative	112	3931.7	0.32	15
4	+1:1000	Positive	>140	1590.0	0.30	19
5	+1:1000	Positive	>140	1315.1	0.35	17
6	+1:320	Positive	42	2967.0	0.39	16
7	+1:100	Positive	21	801.0	0.76	14
8	+1:640	Positive	29	2081.1	0.64	18
9	+1:640	Positive	54	1011.3	0.91	16
10	+1:640	Positive	58	1602.7	0.74	14

ANA = antinuclear antibodies, ESR = erythrocyte sedimentation rate, SLEDAI = system lupus erythematosus disease activity index. The table displayed clinical features including ANA, anti-dsDNA, ESR, 24 h urinary protein, complement C3, and SLEDAI of the systemic lupus erythematosus patient with osteonecrosis at every hospitalization.

**Figure 1:**
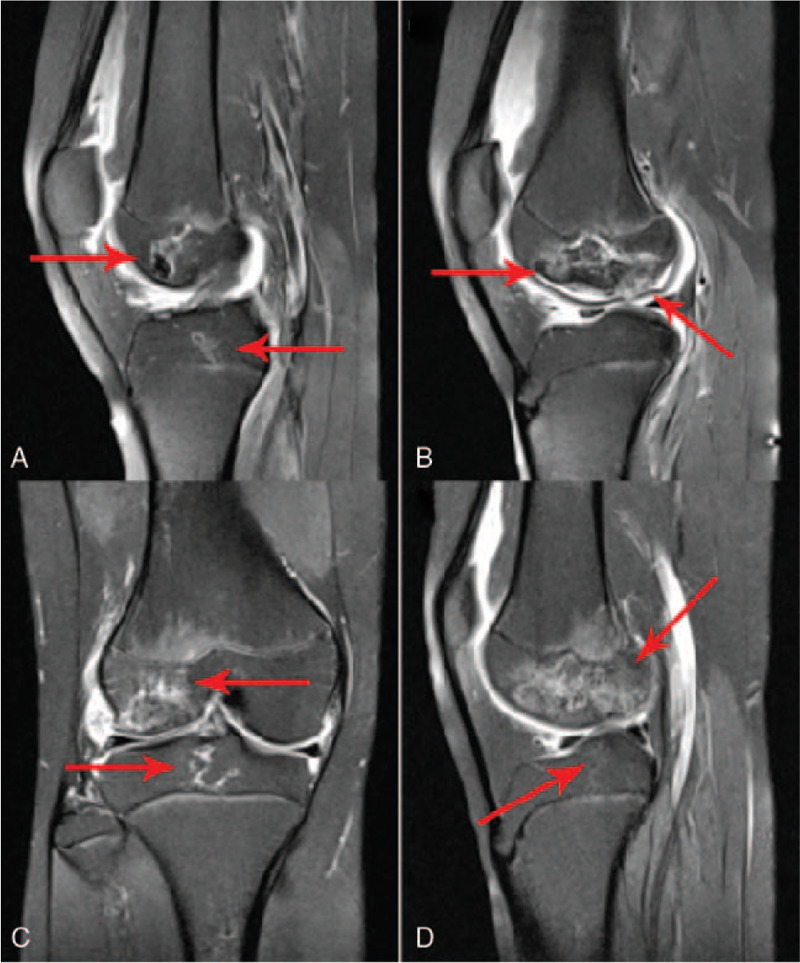
Bilateral knee joint magnetic resonance imaging appearance of the systemic lupus erythematosus patient with osteonecrosis (A) and (B) Sagittal magnetic resonance imagings of left knee joint. (C) and (D) Sagittal magnetic resonance imaging of right knee joint. The figure demonstrated irregular bone destruction and bone hyperplasia lesions on bilateral distal femur and proximal tibia, presenting geographic alterations.

**Figure 2:**
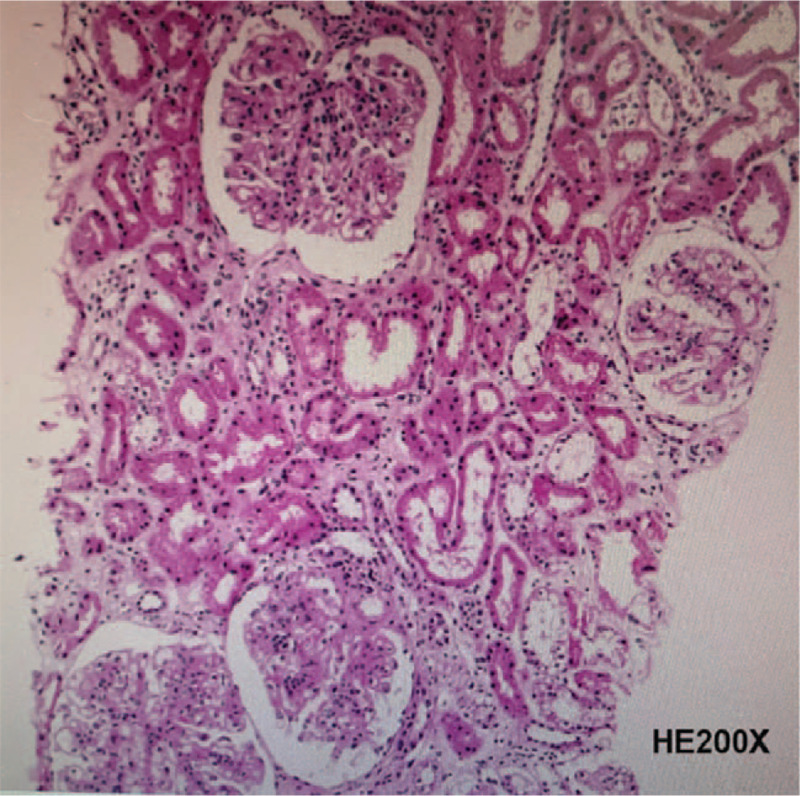
Renal pathological histology of the systemic lupus erythematosus patient with osteonecrosis. Frozen-section showed IgG+++ IgA+++ IgM++ C3+++ C4++ C1q++ Fib+ granular deposition in the capillary loops and mesangium. The renal pathologic diagnosis was in accordance with class IV diffuse proliferative lupus nephritis [IV-G(A/C)].

**Figure 3:**
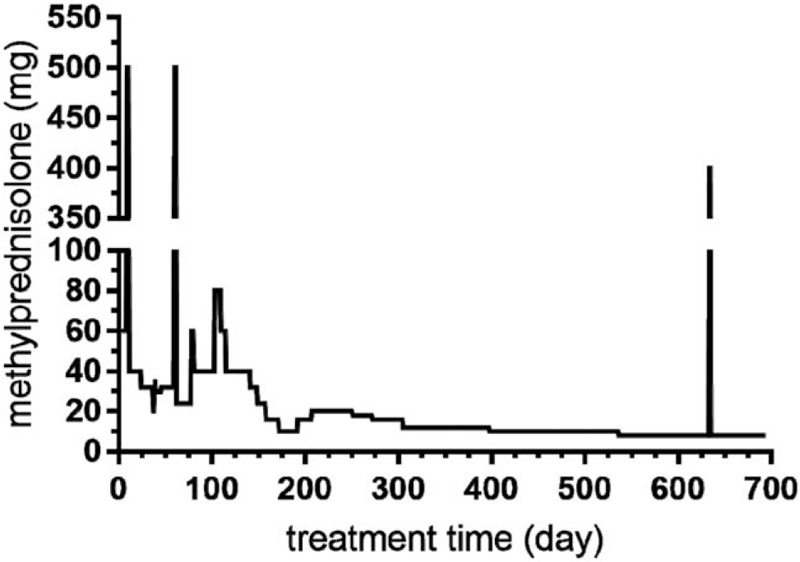
Methylprednisolone dosage during the treatment of the systemic lupus erythematosus patient with osteonecrosis. The figure displays methylprednisolone dosage during the treatment of the systemic lupus erythematosus patient in our case. High-dose methylprednisolone pulse therapies were given 3 times, respectively at the 9th day, 60th day, and 633th day since treatment started. Treatment time of corticosteroids exceeding 20 mg was for more than 200 d.

Nevertheless, in July 2019, when the patient was 13.3 years old (20 months later after diagnosis), she was admitted to the hospital (8th hospitalization) because of a sudden aggravation of sore knees. She suffered from bilateral knee joint pain and her left knee joint manifested typical infection reactions including redness, swelling, heat, and pain. Knee joint MRI screening presented bone destruction and osteoproliferation at the bilateral distal femur and proximal tibia, which implied symptomatic osteonecrosis (Fig. 1). In addition, increased ANA titer, 24-hour urinary protein quantity, and SLEDAI indicated a relapse. Her renal pathologic diagnosis was class IV diffuse proliferative lupus nephritis [IV-G(A/C)] through a percutaneous renal biopsy (Fig. 2). The SLE patient developed ON within 2 years of corticosteroids therapy and a cumulative corticosteroids dose of approximately 12.8 g. During the period of treatment, time of glucocorticoids therapy exceeding 20 mg was for more than 200 days (Fig. 3). After multidisciplinary consultations, the patient finally received high-dose methylprednisolone (0.4 g daily for 2 days) for the third time and multiple intravenous cyclophosphamide (350 mg daily for 2 days) pulse therapies. Meanwhile, the patient was started on oral calcitriol (0.25 μg daily) therapy. In addition, antiplatelet medication dipyridamole (75 mg daily) was taken as adjuvant therapy to prevent thrombus formation. The patient's symptoms alleviated more quickly than expected. Three weeks later, the swelling in her left knee subsided. Her self-reporting pain score decreased from 9 to 4 and walking time increased from 45 minutes to 90 minutes per day. Nearly 5 weeks later, the pain in double knee joint disappeared and the girl could walk without difficulty. At the present stage, surgical interventions are unnecessary for the patient. Alendronate has been recruited into our next therapeutic plan. Now the patient is still undergoing long-term follow-up and treatment.

## 3 Discussion

Osteonecrosis is a known complication of SLE, which mainly affects female SLE patients of childbearing age. Our case presented a 13.3-year-old female SLE patient who developed juvenile multifocal ON with bilateral knee joint involvement. Compared with adolescent and adult SLE patients, pediatric patients have remarkably lower rates of ON. Multifocal ON, with an estimated morbidity of 3% in SLE patients, is rare in juvenile-onset subjects. To our knowledge, previous studies concerning juvenile-onset SLE patients with ON are very limited. The first reported pediatric SLE patients developing ON can be dated back to 1974, in an article published by Hurley et al.^[11]^ Among the 4 patients with avascular necrosis, the youngest was 14 years old when ON was diagnosed in her left hip. In 2010, Nakamura et al^[2]^ investigated 18 pediatric SLE patients (<15 years old) and 25 adolescent SLE patients (15–20 years old), and reported the youngest patients with ON in the hip and knee was 14.9 and 15.5 years old, respectively. They held the view that ON did not develop in patients younger than 14 years old. In 2015, Gurion et al^[3]^ followed up 201 pediatric SLE patients for 36 months and 17 subjects developed or had a history of avascular necrosis. The average age of ON subjects was 16.5 years old and 8 subjects had developed multifocal involvement. Owing to the lack of detailed individual data, the youngest multifocal ON subject was unknown in the study. Based on the existing data, the patient in our case may be the youngest SLE patient who developed multifocal ON. Compared with previous reported juvenile SLE patients, our case suggested a younger age tendency of ON onset and multifocal involvements at the first attack among the other reported pediatric cases.

Corticosteroids are widely applied as the first-line treatment of SLE, and their long-term exposure is closely associated with progress of ON.^[12–15]^ According to a meta analysis, each 10 mg per day increase of corticosteroids was associated with a 3.6% increase in ON occurrence. SLE patients treated with corticosteroids greater than 20 mg per day demonstrated significantly higher odds of ON than those treated with less than 20 mg per day.^[15]^ But corticosteroids are not the only etiology for developing ON. The underlying mechanisms of ON in SLE are linked with internal and external complicated risk factors. Firstly, multiple factors associated with SLE disease itself can contribute to the development of ON. Arthritis, cushingoid, gastrointestinal involvement, hypertension, pleuritis, renal disease, and vasculitis were observed with ON in SLE patients.^[12]^ Additionally, hypercoagulable state and antiphospholipid antibodies which are closely associated with thrombogenesis, can participate in the pathogenesis of ON in SLE.^[16,17]^ Furthermore, researchers found SLE disease activity was a sensitive predictor of ON. Patients with SLEDAI ≥ 8 were significantly at a higher risk of suffering from ON.^[18]^ Secondly, cytotoxic drugs are also a risk factor of ON.^[12,19]^ Long-term high cumulative corticosteroids dose and immunosuppressant have a synergistic effect on the development of avascular necrosis in SLE patients.^[19]^ Thirdly, recent studies revealed gene polymorphisms may increase the risk of ON in SLE patients. Nitric oxide synthase 3 (NOS3) and complement receptor type 2 (CR2) gene polymorphisms, single nucleotide polymorphisms (SNPs) of the adenosine triphosphate-binding cassette B1 (ABCB1) gene were significantly associated with risk of femoral head osteonecrosis in SLE.^[20–22]^ Fourthly, deficiency of important immunoregulatory mediator Vitamin D, has been found to be associated with avascular ON in a pediatric lupus erythematosus trial.^[3]^ Therefore, interaction of multiple risk factors in our case jointly promoted the formation of ON.

There existed limitations during the treatment of our SLE patient. The patient in our case started oral calcitriol therapy after her knee joint ON was diagnosed. After taking medicine, her Vitamin D deficiency improved and her symptomatic ON was relieved. Meanwhile, antiplatelet aggregation drug dipyridamole also played an important role in the inhibition of thrombi formation and ON progress. Alendronate has been recruited into our next therapeutic plan as well. It has been confirmed that alendronate can effectively alleviate pain and delay the progress of ON, avoiding surgical interventions.^[23]^ A recent Japanese study discovered early use of alendronate have a preventive effect against bone loss in corticosteroids-treated juvenile-onset rheumatic diseases.^[24]^ These measures are beneficial for slowing ON deterioration and avoiding surgeries. However, long-term corticosteriods and immunosuppressors, multiple pulse therapies are still used to control active lupus and nephritis. As discussed before, corticosteroids and immunosuppressors together promote the development of ON, which is contradictory to the intention of preventing ON progress. The method of balancing corticosteriods and immunosuppressors in the treatment of SLE and prevention of ON is a challenging problem and deserves further exploration.

Several novel methods for ON prevention and treatment have been widely studied. Anti-coagulant ingredient warfarin and hypolipidemic drug statins can significantly decrease the risk of ON induced by steroids.^[25–27]^ In addition, physical therapies such as extracorporeal shockwave therapy (ESWT), hyperbaric oxygen therapy, and pulsed electromagnetic therapy have been proved to stimulate neovascularization, increase intracellular oxygen, and reduce inflammation reactions in ON lesions.^[28–31]^ Implantation of autologous bone marrow mesenchymal stem cells is a promising treatment of osteonecrosis of femoral head, significantly reducing the risk of femoral head collapse and the need of total hip replacement.^[32–34]^

In summary, the present case report described a 13.3-year-old female SLE patient who developed multifocal ON in her bilateral knee joint. We believe that this case report and the literature review on the underlying mechanisms of ON in SLE patients and the relevant therapeutic methods and preventive measures would help the physicians and pediatricians to have more comprehensive and profound understanding of pathogenesis and treatments of ON in SLE.

## Acknowledgments

We are grateful for the assistance from Department of Rheumatology Immunology and Allergy, Children's Hospital, Zhejiang University School of Medicine in data collection, and the participation of the systemic lupus erythematosus patient in our study.

## Author contributions

**Investigation:** Wenyuan Jin, Xinghui Yang, Meiping Lu.

**Visualization:** Xinghui Yang.

**Writing – original draft:** Wenyuan Jin.

**Writing – review & editing:** Meiping Lu, Wenyuan Jin.

## Abbreviations

Abbreviations: ABCB1 = adenosine triphosphate-binding cassette B1, ANA = antinuclear antibodies, CR2 = complement receptor type 2, ESR = erythrocyte sedimentation rate, ESWT = extracorporeal shockwave therapy, MRI = magnetic resonance imaging, NOS3 = nitric oxide synthase 3, ON = osteonecrosis, SLE = systemic lupus erythematosus, SLEDAI = system lupus erythematosus disease activity index, SNPs = single nucleotide polymorphisms, VEGF = vascular endothelial growth factor.
